# The reciprocal interaction between tumor cells and activated fibroblasts mediated by TNF-α/IL-33/ST2L signaling promotes gastric cancer metastasis

**DOI:** 10.1038/s41388-019-1078-x

**Published:** 2019-10-28

**Authors:** Quan Zhou, Xiongyan Wu, Xiaofeng Wang, Zhenjia Yu, Tao Pan, Zhen Li, Xinyu Chang, Zhijian Jin, Jianfang Li, Zhenggang Zhu, Bingya Liu, Liping Su

**Affiliations:** 1 Department of Surgery, Shanghai Key Laboratory of Gastric Neoplasms, Shanghai Institute of Digestive Surgery, Ruijin Hospital, Shanghai Jiao Tong University School of Medicine, 200025 Shanghai, People’s Republic of China; 2 Department of Urology, Center for Organ Transplantation, Ruijin Hospital, Shanghai Jiao Tong University School of Medicine, 200025 Shanghai, People’s Republic of China; 3 Department of General Surgery, Shanghai General Hospital, Shanghai Jiao Tong University School of Medicine, 200025 Shanghai, People’s Republic of China

**Keywords:** Cancer microenvironment, Mechanisms of disease, Gastric cancer

## Abstract

Gastric cancer (GC) is characterized by extensive local invasion, distant metastasis and poor prognosis. In most cases, GC progression is associated with aberrant expression of cytokines or activation of signaling cascades mediated by tumor–stroma interactions. However, the mechanisms by which these interactions contribute to GC progression are poorly understood. In this study, we find that IL-33 and its receptor ST2L are upregulated in the human GC and served as prognostic markers for poor survival of GC patients. In a co-culture model with GC cells and cancer-associated fibroblasts (CAFs), we further demonstrate that CAFs-derived IL-33 enhances the migration and invasion of GC cells by inducing the epithelial–mesenchymal transition (EMT) through activation of the ERK1/2-SP1-ZEB2 pathway in a ST2L-dependent manner. Furthermore, the secretion of IL-33 by CAFs can be induced by the proinflammatory cytokines TNF-α that is released by GC cells via TNFR2-NF-κB-IRF-1 pathway. Additionally, silencing of IL-33 expression in CAFs or ST2L expression in GC cells inhibits the peritoneal dissemination and metastatic potential of GC cells in nude mice. Taken together, these results characterize a critical role of the interaction between epithelial-stroma mediated by the TNF-α/IL-33/ST2L signaling in GC progression, and provide a rationale for targeting this pathway to treat GC metastasis.

## Introduction

Gastric cancer (GC), the second leading cause of cancer death worldwide, is known to have a dismal prognosis due to its predisposition for invasion and metastasis [1–5]. Rapid progression of GC without specific clinical symptoms makes this disease challenging to diagnosis and treatment in the early stage, which may contribute to its high mortality. Surgical resection remains the main treatment for the disease, however, the overall prognosis for patients with non-resected GC is dismal. Therefore, it is important to elucidate the molecular pathogenesis involved in GC and identify novel prognostic markers and therapeutic strategies [6, 7].

Accumulating evidence indicates that cross-talk between tumor cells and stromal cells plays a critical role in promoting tumor invasion and metastasis [8–13]. Cancer-associated fibroblasts (CAFs), the activated fibroblasts in cancer stroma, have been shown to promote cancer progression by interacting with diverse cell types through the secretion of cytokines and local extracellular matrix (ECM) [14–16]. We previously demonstrated that CAFs secrete HGF to promote GC tumorigenesis in a paracrine manner [17]. However, more likely, the cross-talk between tumor cells and stromal cells is to be multi-factorial, involving multiple cytokines, cell-specific receptors and intracellular signaling pathways. Interleukin-33 (IL-33), a member of the IL-1 superfamily, is a multifunctional cytokine that serves as a “danger signal” and is released upon inflammatory response, biomechanical stress or necrotic cell death [18–20]. IL-33 is predominantly expressed in various types of cells, including dendritic cells and macrophages, epithelial cells, stromal fibroblasts and endothelial cells. IL-33 has been found to mediate Th2 immune responses by binding and signaling through ST2L, an orphan receptor in the IL-1R family, to induce NF-κB and MAPK (p38, JNK and ERK1/2) activation, and promote Th2 cytokine production [19–23]. Recent studies have revealed that IL-33 plays a functional role in tumor development, with high levels of serum IL-33 associated with poor prognosis and chemotherapy resistance in various cancers [24–30]. An IL-33-dependent proliferation response can be induced by the increase and recruitment of type 2 innate lymphoid cells (ILC2s) [31], which produce high levels of IL-13 that consequently promote cholangiocyte hyperplasia [32]. IL-33 can stimulate the production of pro-inflammatory IL-6 and pro-angiogenic IL-8 and may function as a critical inducer in inflammation-associated pancreatic carcinogenesis [33]. Circulating IL-33 levels have also been shown to be significantly increased in GC patients and to be closely correlated with the depth of invasion, advanced stage and distant metastasis [34]. However, the mechanisms underlying the regulation of IL-33 expression in stromal fibroblasts and its effect on GC progression remain largely uncharacterized.

In the present study, we find that both IL-33 in stromal cells and the IL-33 receptor ST2L in tumor cells are aberrantly overexpressed in GC and serve as prognostic markers for poor survival. Using an in vitro model, we demonstrate that CAFs-derived IL-33 promotes migration, invasion and epithelial–mesenchymal transition (EMT) of GC cells by activation of the ERK1/2/SP1/ZEB2 pathway via ST2L, and that in turn, GC cell derived-TNF-α upregulates IL-33 expression in CAFs via the TNFR2/NF-κB/IRF-1 pathway. Furthermore, silencing IL-33 expression in CAFs or ST2L expression in GC cells can significantly inhibit the metastatic potential of GC cells in vivo. Thus, we identify TNF-α/IL-33/ST2L signaling as a mediator of the tumor–stromal cell interaction in GC.

## Results

### IL-33 and its receptor ST2L are overexpressed in human GC and predict poor prognosis

**Fig. 1:**
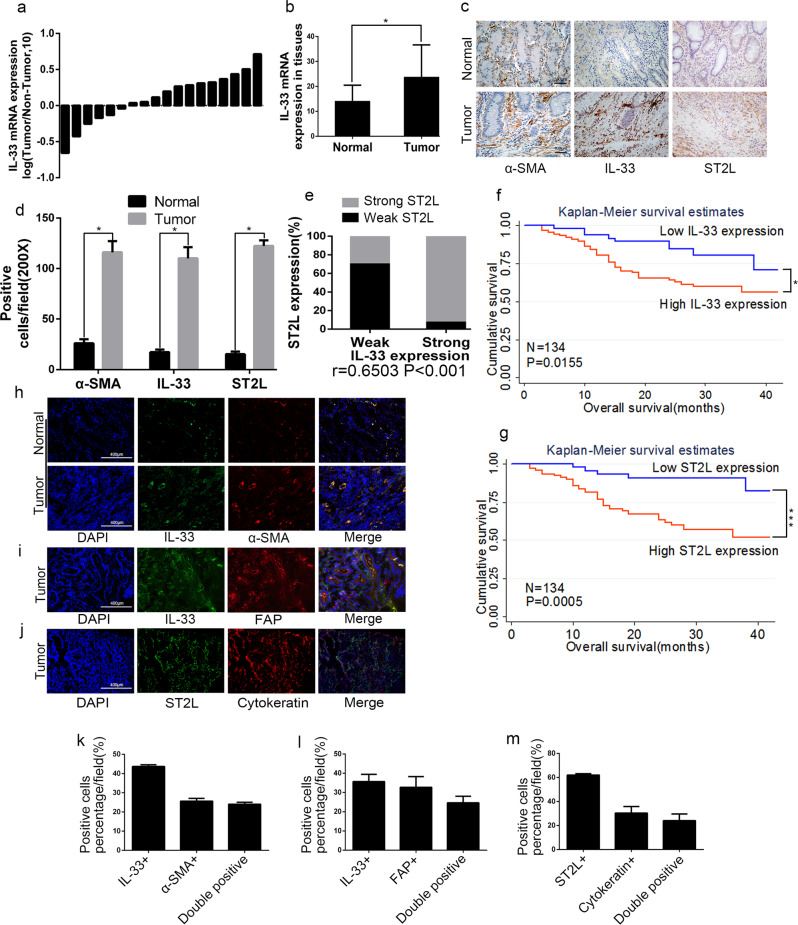
IL-33 and ST2L are overexpressed in human GC tissues and predict poor prognosis. **a** QRT-PCR analysis of *IL-33* mRNA expression in GC and corresponding normal tissues (*n* = 18). **b** Histogram displaying the relative mRNA expression of *IL-33* in 18 GC tissues. Data are shown as −ΔΔCt and 2^−ΔCt^. **c** IHC staining of α-SMA, IL-33 and ST2L in GC tissues (200×; scale bar = 100 μm). **d** Histogram displaying the number of α-SMA, IL-33 and ST2L positive cells/field in GC tissues. **e** Histogram displaying the correlation between IL-33 expression and ST2L expression determined by IHC (*r* = 0.6503). **f**, **g** Survival of patients with low or high levels of IL-33 and ST2L protein expression. **h**–**j** Immunofluorescence staining of IL-33, α-SMA, FAP, ST2L and Cytokeratin in GC tissues (200×; scale bar = 400 μm). **k**–**m** Histogram displaying the percentage of IL-33^+^/α-SMA^+^, IL-33^+^/FAP^+^ and ST2L^+^/Cytokeratin^++^ cells in GC tissues. Data are represented as the mean ± SD; *P <0.05, **P <0.01, ***P <0.001

To elucidate the role of IL-33 and its receptor ST2L in human GC, we first quantified their expression levels and cellular source in GC tissues. The expression of IL-33 mRNA was quantified by Quantitative Real-time PCR (QRT-PCR) in 18 pairs of GC tissues and corresponding non-cancerous tissues. We found that the expression level of IL-33 mRNA was elevated in GC tissues in comparison with non-cancerous tissues (Fig. 1a, b). We further analyzed the protein levels of IL-33 and ST2L in 134 pairs of GC tissues and corresponding non-cancerous tissues by immunohistochemistry (IHC). As a control, α-SMA that is known to be overexpressed in CAFs within GC stroma [17] was also assessed. As shown in Fig. 1c, IL-33, ST2L and α-SMA were strongly expressed in GC tissues compared to normal tissues. Through the staining for α-SMA, IL-33 was primarily restricted to the stroma, whereas the staining for ST2L was primarily restricted to the epithelial cells. The positive rates of IL-33 expression were 64.2% (86/134) in GC tissues and 35.8% (48/134) in non-cancerous tissues (Fig. 1c), and the number of IL-33-positive cells was positively correlated with that of α-SMA-positive cells (Fig. 1d). Moreover, ST2L expression in epithelial cells positively correlated with IL-33 expression in stromal cells (*r* = 0.6503, *P* < 0.001) (Fig. 1c, e), which suggested that IL-33 and its receptor ST2L, while being expressed in different cell types, were coordinately overexpressed in the same GC tissues. To assess the clinical significance of the elevated IL-33 and ST2L in GC, we performed multivariate statistical analysis. Table 1 showed that the expression levels of IL-33 and ST2L protein were significantly associated with local invasion (*P* < 0.001) and Tumor-Node-Metastasis (TNM) stage (*P* < 0.05). Kaplan–Meier survival analysis revealed that GC patients with high IL-33 or ST2L expression had a shorter overall survival (Fig. 1f, g).

**Table 1:** Relationship between IL-33 and ST2L expression levels and clinicopathologic parameters in 134 GC tissues

Variables	Number of cases	IL-33 immunostaining		*P* value	ST2L immunostaining		*P* value
		Weak	Strong		Weak	Strong	
Age (years)							
≤60	59	18	41	0.281	14	45	0.136
>60	75	30	45		27	48	
Gender							
Male	95	36	59	0.552	32	63	0.303
Female	39	12	27		9	30	
Tumor size (cm)							
≤5	62	19	43	0.281	17	45	0.573
>5	72	29	43		24	48	
Lauren classification							
Intestinal	74	24	50	0.372	19	55	0.372
Diffuse	60	24	36		22	38	
Differentiation							
Poorly, undifferentiated	83	30	53	1.000	27	56	0.568
Well, moderately	51	18	33		14	37	
Local invasion							
T1, T2	33	21	12	<0.001	20	13	<0.001
T3, T4	101	27	74		21	80	
Lymph node metastasis							
No	44	18	26	0.445	9	35	0.11
Yes	90	30	60		32	58	
TNM stage							
I, II	29	18	11	0.002	15	14	0.011
III, IV	105	30	75		26	79	

To further identify cellular sources that are responsible for the higher expression of IL-33 and ST2L in GC tissues, we detected the co-expression of FAP (the marker of CAFs) and IL-33, Cytokeratin (the marker of epithelial cells) and ST2L in GC tissues by immunofluorescence (IF). As shown in Fig. 1h–j, IL-33 was strongly expressed in CAFs as identified by anti-α-SMA or anti-FAP antibody staining, while ST2L was mainly expressed in GC cells as identified by anti-Cytokeratin antibody staining. Double-positive (IL-33^+^/α-SMA^+^, IL-33^+^/FAP^+^ and ST2L^+^/Cytokeratin^+^) cells account for a high percentage of the total cells that are positive for α-SMA, FAP or Cytokeratin in GC tissues (Fig. 1k–m). Consistently, the expression of IL-33 mRNA and protein was significantly higher in CAFs compared to seven GC cell lines, while the protein expression of ST2L was elevated in most of the GC cell lines compared with CAFs and normal fibroblasts (NFs) (Fig. S1a–c). Thus, these findings indicated that IL-33 and ST2L are coordinately overexpressed CAFs and GC cells of human GC tissues and predict poor prognosis.

### CAFs-derived IL-33 promotes the migration, invasion and EMT of GC cells via ST2L

**Fig. 2:**
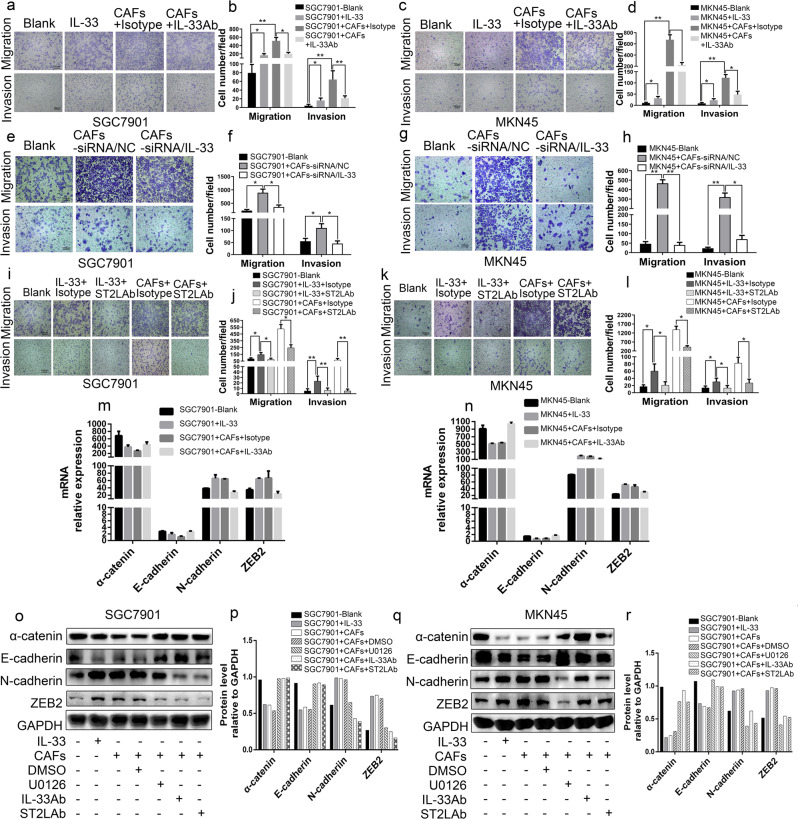
CAFs-derived IL-33 promotes the migration, invasion and EMT of GC cells. **a**–**d** The migration and invasion ability of SGC7901 and MKN45 cells were analyzed after culture in medium alone (blank) or treatment with: exogenous IL-33 (300 ng/ml); co-culture with CAFs supplemented with IgG isotype antibody (3 μg/ml) or IL-33 neutralizing antibody (3 μg/ml). Histograms show the average cell number/field (100×; scale bar = 100 μm). **e**–**h** The migration and invasion ability of SGC7901 and MKN45 cells was detected after culture in medium alone (blank), co-culture with CAFs transfected with IL-33/siRNA or nc/siRNA. Histograms display the average cell number/field (100×; scale bar = 100 μm). **i**–**l** The migration and invasion ability of SGC7901 and MKN45 cells was analyzed after culture in medium alone (blank) or stimulation with exogenous IL-33 (300 ng/ml) plus IgG isotype antibody or ST2L neutralizing antibody (3 μg/ml); co-culture with CAFs in the presence of IgG isotype antibody (3 μg/ml) or ST2L neutralizing antibody (3 μg/ml). Histograms show the average cell number/field. (100×; scale bar = 100 μm). **m**, **n** QRT-PCR of the genes for EMT in SGC7901 and MKN45 cells after culture in medium alone (blank); or activation with exogenous IL-33 (300 ng/ml); co-culture with CAFs in the presence of IgG isotype antibody (3 μg/ml) or IL-33 neutralizing antibody (3 μg/ml). **o**–**r** Western blot of EMT markers in SGC7901 and MKN45 cells cultured in medium alone (blank); or stimulated with exogenous IL-33 (300 ng/ml) or co-culture with CAFs or co-cultured with CAFs in the presence of DMSO, U0126 (20 μM), IL-33 neutralizing antibody (3 μg/ml) or ST2L neutralizing antibody (3 μg/ml). **p** and **r** Densitometric analysis shows the expression level of EMT markers in GC cells stimulated by the above factors. Data are represented as the mean ± SD of three independent experiments; *P <0.05, **P <0.01, ***P <0.001

To investigate the role of CAFs-derived IL-33 in GC progression, CAFs expressing high levels of IL-33 were co-cultured with SGC7901 and MKN45 cells that expressed moderate levels of ST2L. Although IL-33 had no effects on the proliferation capability of SGC7901 and MKN45 cells (data not shown), the addition of exogenous IL-33 or co-culture with CAFs led to increased migration and invasion of both SGC7901 and MKN45 cells, while anti-IL-33 antibody attenuated these stimulatory effects of CAFs on GC cells (Fig. 2a–d). We further determined whether knockdown of IL-33 could also impair GC cell migration and invasion triggered by CAFs in vitro. To establish an IL-33 loss-of-function model, CAFs were transfected with human IL-33 siRNA, and IL-33 downregulation was validated by QRT-PCR and enzyme-linked immunosorbent assay (ELISA) (Fig. S2a, b). As shown in Fig. 2e–h, IL-33 silencing in CAFs significantly decreased the number of migrated or invaded GC cells (Fig. 2e–h). These results verified the role of CAFs-derived IL-33 in mediating GC cell migration and invasion.

To further determine ST2L on GC cells as the receptor for IL-33 activation of migration and invasion, we repeated Transwell migration and invasion assays in the presence of neutralizing ST2L antibody or an isotype control antibody. Neutralization of ST2L led to significantly decreased migration and invasion of GC cells as induced either by exogenous IL-33 or CAFs co-culture (Fig. 2i–l). Because ST2L was expressed predominately on GC cells (Fig. S1c), these results suggested that CAFs-derived IL-33 promoted GC cell migration and invasion through ST2L.

EMT is a critical process in cancer metastasis and plays an important role in GC cells migration and invasion. To further explore potential mechanism regulating the malignant effects of CAFs-derived IL-33 on GC cells, we next investigated whether the IL-33 status of CAFs could regulate EMT of GC cells. The addition of exogenous IL-33 or co-culture with CAFs markedly decreased the expression of the epithelial cell markers α-catenin and E-cadherin while increasing the expression of the mesenchymal cell markers N-cadherin and ZEB2 in both SGC7901 cells and MKN45 cells; however, IL-33 neutralizing antibody significantly reversed this effect (Fig. 2m, n). Moreover, the EMT of GC cells induced by CAFs co-culture was reversed partially or fully by the addition of the specific ERK1/2 inhibitor U0126, IL-33 neutralizing antibody, or ST2L neutralizing antibody (Fig. 2o–r). Collectively, these results demonstrated that CAFs-derived IL-33 promotes the migration, invasion and EMT of GC cells via ST2L, with a potential role of the ERK1/2 signaling pathway.

### The metastatic potential of GC cells induced by CAFs-derived IL-33 is mediated by the activation of ERK1/2-SP1-ZEB2 pathway via ST2L

**Fig. 3:**
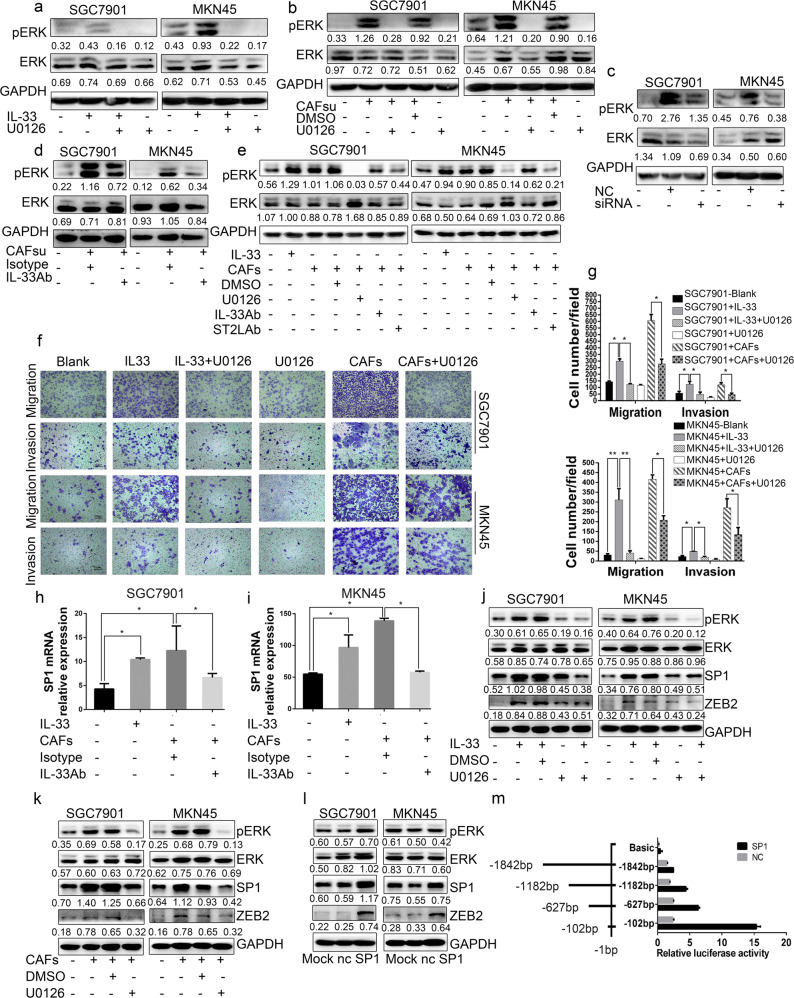
The metastatic potential of GC cells induced by CAFs-derived IL-33 is mediated by the activation of ERK1/2-SP1-ZEB2 pathway via ST2L. **a**–**d** Western blot analysis showing the phosphorylation of ERK1/2 in SGC7901 and MKN45 cells treated with medium alone or stimulated as follows: exogenous IL-33 (300 ng/ml), U0126 (20 μM), CAFs supernatants (CAFsu) supplemented with IgG isotype control antibody (3 μg/ml), DMSO; co-cultured with CAFs transfected with IL-33/siRNA or NC/siRNA; CAFsu supplemented with IL-33 neutralizing antibody (3 μg/ml). **e** Western blot analysis of pERK1/2 in SGC7901 and MKN45 cells incubated with medium alone; exogenous IL-33 (300 ng/ml); U0126 (20 μM); co-culture with CAFs; IL-33 neutralizing antibody (3 μg/ml); and/or ST2L neutralizing antibody (3 μg/ml). **f**, **g** The migration and invasion ability of SGC7901 and MKN45 cells after culture in medium alone (blank) or stimulation with exogenous IL-33 (300 ng/ml) and/or U0126 (20 μM); co-culture with CAFs and/or U0126 (20 μM). Histograms show the average cell number per field. (100×; scale bar = 100 μm). **h**, **i** QRT-PCR analysis of *SP1* mRNA expression in SGC7901 and MKN45 cells incubated in medium alone or stimulation with exogenous IL-33 (300 ng/ml) or CAFs in the presence of IgG isotype control antibody (3 μg/ml) or IL-33 neutralizing antibody (3 μg/ml). **j**, **k** Western blot analysis of pERK1/2, ERK1/2, SP1 and ZEB2 in SGC7901 and MKN45 cells incubated in medium alone or stimulated by co-culture with CAFs in the presence of exogenous IL-33 (300 ng/ml) or U0126 (20 μM). **l** Western blot analysis showing the protein expression of pERK1/2, ERK1/2, SP1 and ZEB2 in SGC7901 and MKN45 cells cultured in medium alone (Mock); or transfected with control plasmid (nc) or SP1-expressing plasmid. **m** Dual luciferase reporters containing four different lengths of the ZEB2 promoter were co-transfected with SP1-expressing plasmid into 293T cells. Firefly luciferase activity was assessed relative to Renilla luciferase activity. Data are representative of three independent experiments. Densitometry shows relative protein expression. *P <0.05, **P <0.01, ***P <0.001

IL-33 binds to ST2L and activates multiple downstream pathways including ERK1/2 that plays an important role in cell migration and invasion [35, 36]. Our initial results indicated that the EMT of GC cells induced by IL-33 was inhibited by U0126 (Fig. 2o–r), we therefore assessed whether CAFs-derived IL-33 could modulate the activation of the ERK1/2 pathway in GC cells. Western blot analysis demonstrated increased ERK1/2 phosphorylation in GC cells after culture with exogenous IL-33 (Fig. 3a) or CAFs (Fig. 3b), and not surprisingly ERK1/2-specific inhibitor U0126 abrogated IL-33 or CAFs-induced ERK1/2 phosphorylation in both SGC7901 cells and MKN45 cells. Moreover, both IL-33 siRNA and IL-33 neutralizing antibody reversed the activation of ERK1/2 in GC cells upon CAFs co-culture (Fig. 3c, d), which verified that the ERK1/2 activation was IL-33-dependent. Notably, anti-ST2L antibody also inhibited the phosphorylation of ERK1/2 in GC cells after co-culture with CAFs (Fig. 3e).

EMT can be triggered by different signals received from the tumor microenvironment. The downregulation of the cell–cell junction protein E-cadherin, which is one of the hallmarks of EMT, can be achieved by ZEB2 through its direct binding to conserved E2 boxes present in the E-cadherin promoter. SP1, a transcription factor that is associated with ERK1/2, has been shown to interact with ZEB2, repress E-cadherin expression and initiate the process of EMT [37–39]. Furthermore, our results suggested that SP1 was highly expressed in GC cells, but not in CAFs (Fig. S3a, b). Thus, we explored whether the ERK1/2-SP1-ZEB2 axis could mediate IL-33-induced EMT in GC cells. Our results demonstrated that SP1 mRNA expression was elevated in SGC7901 and MKN45 cells after treatment with IL-33 or co-culture with CAFs, while deprivation of IL-33 with anti-IL-33 neutralizing antibody significantly reversed this effect (Fig. 3h, i). Moreover, the activation of SP1 and ZEB2 upon treatment with exogenous IL-33 (Fig. 3j) or CAFs co-culture (Fig. 3k) could be inhibited by U0126, which confirmed that their activation incurred downstream of IL-33-mediated ERK1/2 activation. To verify that ZEB2 is activated by SP1, we overexpressed SP1 in SGC7901 and MKN45 cells. SP1 significantly enhanced the protein expression of ZEB2 but had no effect on ERK1/2 activation (Fig. 3l), which suggested that SP1 is activated downstream of ERK1/2 signaling pathway and upstream of ZEB2. To further elucidate the precise mechanism by which SP1 promotes the expression of ZEB2 in GC cells, we examined the ZEB2 promoter region for potential SP1-binding sites [40], and found four putative sites with relatively high scores (Fig. S3c, d). Then, firefly luciferase reporter-gene plasmids expressing full length or truncated ZEB2 promoters were constructed. The relative luciferase activity was higher in SP1-transfected 293T cells than that in control cells (NC) when co-transfected with the ZEB2 promoter (−102 bp, −627 bp, −1182 bp, −1842 bp) (Fig. 3m), which indicated that SP1 upregulates ZEB2 expression by binding its promoter.

In consistent with this, ERK1/2 specific inhibitor U0126 significantly inhibited cell migration, invasion and EMT of both SGC7901 cells and MKN45 cells induced by exogenous IL-33 and CAFs (Figs. 3f, g, 2o–r). In addition, we inhibited SP1 activity by Mithramycin A or depleted ZEB2 by siRNA in GC cells, and then analyzed EMT markers after stromal IL-33 treatment. While control cells underwent marked EMT after stromal IL-33 treatment, which was demonstrated with decreased α-catenin, E-cadherin and increased the N-cadherin, SP1-inhibited cells and ZEB2-depleted cells did not acquire EMT characteristics (Fig. S4). Taken together, these findings indicated that CAFs-derived IL-33 regulates GC cells migration, invasion and EMT via ST2L-ERK1/2-SP1-ZEB2 pathway, which contributes to the metastasis potential of GC cells.

### GC cell-derived TNF-α promotes IL-33 expression in CAFs via the TNFR2-NF-κB-IRF-1 pathway

**Fig. 4:**
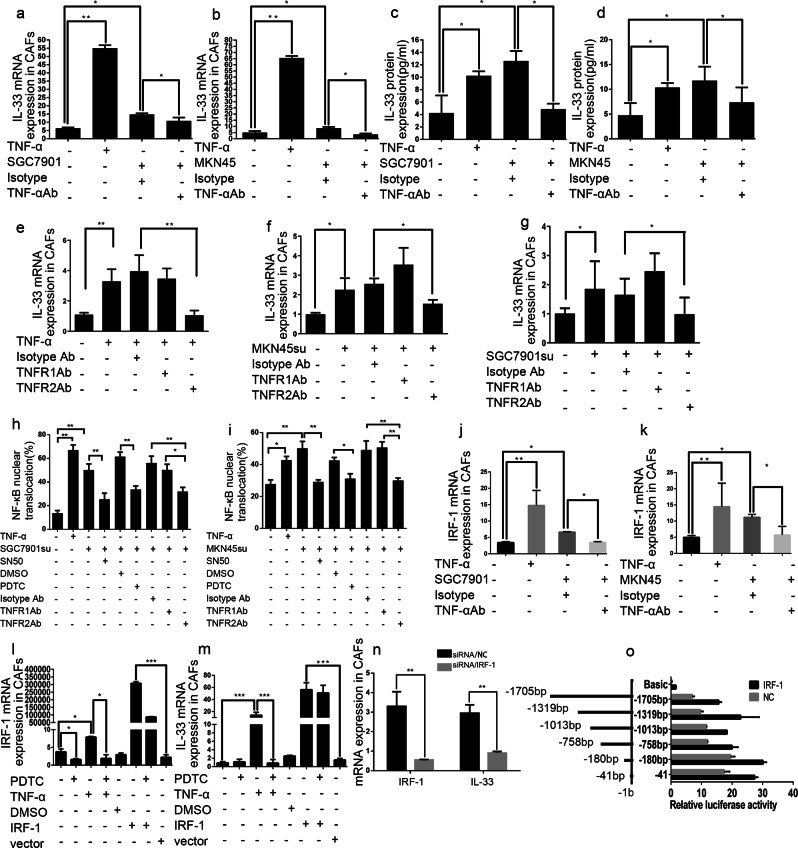
GC cell-derived TNF-α promotes the expression of IL-33 in CAFs. **a**, **b** *IL-33* mRNA expression by CAFs was assessed by QRT-PCR after culture in medium or activation with the following stimuli: exogenous TNF-α (50 ng/ml); co-culture with SGC7901 or MKN45 cells; IgG isotype antibody (3 μg/ml); TNF-α neutralizing antibody (3 μg/ml). **c**, **d** IL-33 protein expression in the supernatants of CAFs was assessed by ELISA after activation as in **a** and **b**. **e**–**g** *IL-33* mRNA expression by CAFs was assessed by QRT-PCR after culture in medium or activation with the following stimuli: exogenous TNF-α (50 ng/ml); supernatants from MKN45 cells (MKN45su) or SGC7901 cells (SGC7901su); isotype antibody (3 μg/ml); TNFR1 neutralizing antibody (10 μg/ml); TNFR2 neutralizing antibody (10 μg/ml). **h**, **i** The nuclear translocation of p65 in CAFs was detected by IF after culture in medium alone or activation with the following stimuli: exogenous TNF-α (50 ng/ml); supernatants from SGC7901cells (SGC7901su) or from MKN45 cells (MKN45su); SN50 (5 μM); PDTC (5 μM); IgG isotype antibody (3 μg/ml); TNFR1 (10 μg/ml) or TNFR2 (10 μg/ml) neutralizing antibodies. Histograms displaying the number of p65 nuclear translocation in each group. **j**, **k** *IRF-1* mRNA expression by CAFs was assessed by QRT-PCR after activation as in **a** and **b**. **l** *IRF-1* mRNA expression by CAFs was assessed by QRT-PCR after culture in medium alone or stimulation with PDTC, TNF-α or DMSO. **m** *IL-33* mRNA expression by CAFs was assessed after activation as in **l**. **n** The mRNA expression of IL-33 and IRF-1 in CAFs was explored by QRT-PCR after IRF-1 siRNA transfection. **o** Dual luciferase reporters containing six different truncations of the IL-33 promoter region were co-transfected with IRF-1-expressing plasmid into 293T cells. Firefly luciferase activity was detected relative to Renilla luciferase activity. Data are representative of three independent experiments. *P < 0.05, **P < 0.01, ***P < 0.001

To unravel the regulatory mechanisms of IL-33 overexpression in GC, we examined the cross-talk between GC cells and CAFs. Paracrine mediators including proinflammatory cytokines and growth factors in tumor microenvironment are critical to the tumor–stroma interactions during tumor progression. We therefore examined the response of CAFs to TNF-α, which is highly expressed in GC tissues and can be produced by GC cells (Fig. S5a–c). As shown in Fig. 4a–d, stimulation with exogenous TNF-α or co-culture with GC cells significantly increased IL-33 expression in CAFs. As expected, this increase was reversed by the addition of neutralizing TNF-α antibody. Thus, these results indicated that TNF-α, produced by GC cells, could promote the secretion of IL-33 by CAFs.

To further elucidate the potential mechanism by which tumor cell-derived TNF-α promotes IL-33 expression in CAFs, we examined the expression of TNF-α receptors (TNFR1 and TNFR2) in both GC cells and CAFs. Both TNFR1 and TNFR2 mRNA were expressed in GC cells and CAFs (data not shown). However, the induced expression of IL-33 in CAFs by TNF-α or co-culture with GC cells was only reduced in the presence of TNFR2 but not TNFR1 neutralizing antibody (Fig. 4e–g). To investigate the molecular mechanisms underlying IL-33 activation in CAFs through TNFR2, we analyzed the promoter of the human IL-33 gene using two online tools [40], and found several putative interferon regulatory factor 1 (IRF-1) binding sites with relatively high scores (Fig. S5d). TNF-α is known to exert its biological effects via TNFRs by enhancing NF-κB activation [41]. Therefore, we hypothesized that TNF-α/TNFR2/NF-κB/IRF-1 pathway may be involved in the regulation of IL-33 expression in CAFs. To examine this hypothesis, we first determined whether GC cell-derived TNF-α could promote the activation of NF-κB in CAFs. We found that exogenous TNF-α or culture with GC cell supernatant could significantly promote the nuclear translocation of p65 in CAFs (Fig. 4h, i, Fig. S6), which indicated the activation of NF-κB pathway. Meanwhile, we used NF-κB specific inhibitor PDTC or SN50 to interrupt the NF-κB signal pathway, and the nuclear translocation level of p65 was strikingly decreased. To confirm NF-κB activation was triggered by TNF-α/TNFR2, we blocked TNFR1 or TNFR2 by neutralizing antibodies respectively, and detected the translocation percentage of p65. Consistent with our prior hypothesis, the increased translocation of p65 was attenuated only in the presence of TNFR2 neutralizing antibody. We then determined whether TNF-α could induce the expression of IRF-1 in CAFs. Our results demonstrated that TNF-α or co-culture with GC cells significantly induced the expression of IRF-1 in CAFs, which was abrogated by TNF-α neutralizing antibody (Fig. 4j, k). Moreover, induction of IRF-1 by TNF-α was abrogated by PDTC (Fig. 4l), suggesting that IRF-1 induction occurs downstream of NF-κB. In addition, transfection with IRF-1 plasmid promoted the expression of IL-33 in the absence of TNF-α, while PDTC blocked the induction of IL-33 by TNF-α but not IRF-1 (Fig. 4m). We then explored the expression of IL-33 and IRF-1 in CAFs transfected with IRF-1 siRNA. As shown in Fig. 4n, IRF-1 knockdown in CAFs significantly decreased the expression of IL-33. Taken together, these findings confirmed that that IRF-1 functions upstream of IL-33 and downstream of NF-κB in the TNF-α pathway.

To further elucidate the mechanisms by which IRF-1 promotes the expression of IL-33 in CAFs, we constructed firefly luciferase reporter-gene plasmids expressing full length or truncated IL-33 promoter. The relative luciferase activity was higher in 293T/IRF-1 cells co-transfected with IL-33 promoter (−41, −180, −758, −1013, −1319, −1705 bp) than that in control cells (NC) (Fig. 4o). Taken together, these results suggested that tumor cell-derived TNF-α promotes the expression of IL-33 in CAFs via the TNFR2/NF-κB/IRF-1 pathway.

### Knockdown of IL-33 expression in CAFs or ST2L expression in GC cells attenuates GC cell peritoneal dissemination and metastasis in vivo

**Fig. 5:**
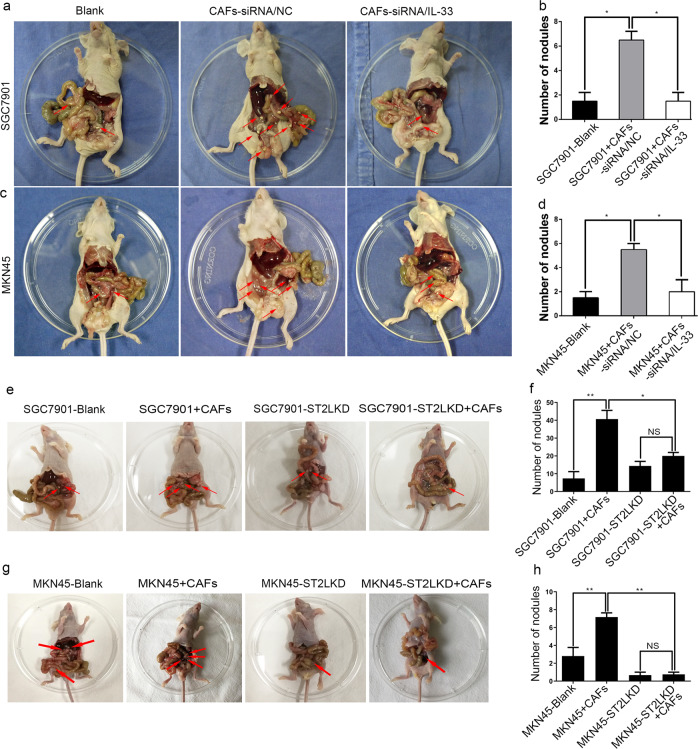
Knockdown of IL-33 expression in CAFs or ST2L expression in GC cells inhibits peritoneal dissemination of GC cells in nude mice. **a**, **c** In vivo tumor peritoneal dissemination was examined by co-injection of SGC7901 or MKN45 cells and CAFs transfected with control siRNA (NC) or IL-33 siRNA into nude mice. Metastatic nodules are indicated by the red arrows. **b**, **d** Histograms displaying the number of peritoneal nodules in nude mice. **e**–**h** In vivo tumor peritoneal dissemination was examined by co-injection of CAFs and GC cells with ST2L knockdown (KD) into nude mice. Metastatic nodules are indicated by the red arrows. **f**, **h** Histograms displaying the number of peritoneal nodules in nude mice. Data are representative of three independent experiments. *P <0.05, *P <0.05, **P <0.01

Peritoneal metastasis from the disseminations of GC cells is the most frequent pattern of GC recurrence or metastasis. We then tested whether the crosstalk between CAFs and GC cells mediated by IL-33/ST2L could actually play a crucial role in promoting GC cells peritoneal dissemination and metastasis in vivo by co-injection of CAFs with GC cells intraperitoneally into nude mice. Gastric CAFs producing high level of IL-33 were transfected with human IL-33 siRNA (siRNA/IL-33) to deprive the secretion of IL-33 (Fig. S2a, b). ST2L expression of GC cells (SGC7901, MKN45) was silenced by CRISPR/Cas9 System. As shown in Fig. 5a–d, knockdown of IL-33 in CAFs resulted in the significantly fewer visible peritoneal nodules in both SGC7901 + CAFs-siRNA/IL-33 group and MKN45 + CAFs-siRNA/IL-33 group compared to SGC7901 + CAFs-siRNA/NC group or MKN45 + CAFs-siRNA/NC group, respectively. Similarly, silencing ST2L expression in SGC7901 or MKN45 cells also generated less peritoneal nodules in nude mice when mixed with CAFs (Fig. 5e–h). Moreover, consistent with the promotion of EMT by CAFs-derived IL-33 in vitro, there were also decreased expression of ZEB2 in peritoneal tumors derived from GC cells mixed with CAFs-siRNA/IL-33 than in tumors derived from GC cells mixed with CAFs-siRNA/NC (Fig. S7). Thus, these data suggest that disruption of the crosstalk between CAFs and GC cells by knockdown of IL-33 expression in CAFs or ST2L expression in GC cells attenuates GC cells peritoneal dissemination and metastasis in vivo.

## Discussion

CAFs, the major stromal cells in tumor microenvironment, play key roles in tumor initiation and metastasis via an intricate interplay of tumor microenvironment components [14, 42]. However, the mechanisms by which CAFs interact with tumor cells are not fully elucidated in GC. In this study, we demonstrated that the aberrant expression of IL-33 and its receptor ST2L predict poor prognosis of GC patients. Gastric CAFs express high level of IL-33, while GC cells express ST2L, which suggest that IL-33/ST2L axis may act as an important mediator of tumor-stromal cell interactions in GC.

CAFs are known to commit an invasive phenotype and tumor-promoting properties. Andersson et al. reported that CAFs producing high level of IL-33 induced the transition of M1 to M2 macrophage, governing stromal-mediated metastasis via IL-33-NF-κB-MMP9-laminin axis [43]. Moreover, the study by Chen et al. indicated that CAFs promoted the migration and invasion of head and neck squamous cell carcinoma (HNSCC) by autocrine and paracrine effects on tumor-microenvironmental IL-33 signaling [44]. We previously identified several key factors in mediating CAFs’ tumor-promoting properties in GC, including HGF, Lumican and IL-6 [17, 45, 46]. Notably, although IL-33 has already been described in the serum of GC patients [34], little information is known about the role of stromal IL-33 in GC. In the present study, we found that CAFs-derived IL-33 promotes the migration, invasion and EMT of GC cells in vitro and in vivo, and these effects are mediated by the activation of ERK1/2 pathway via ST2L. We also demonstrated that EMT, a critical cellular process to cancer metastasis, is induced by stromal IL-33. SP1 is a key regulator in mediating peritoneal cancer dissemination and niche-directed metastasis [47]. CAFs-derived IL-33 increases SP1 expression in GC cells, which can be attenuated by the ERK1/2 inhibitor U0126; SP1 upregulates ZEB2 expression by binding its promoter, and the inhibition of SP1 is sufficient to prevent ZEB2 expression and EMT. Thus, we uncovered a novel mechanism by which CAFs-derived IL-33 promotes GC progression by inducing GC cell EMT via the ST2L-ERK1/2-SP1-ZEB2 pathway.

Endogenous factors released by tumor cells or present in the tumor microenvironment might serve as endogenous stimuli for NF-κB, which is required for proinflammatory cytokine activation and release. Proinflammatory CAFs in a transgenic mouse tumor model were shown to promote inflammation in a NF-κB-dependent manner [48]. IL-33 is highly expressed in GC stroma, but the molecular mechanism regulating IL-33 expression remains unclear [27, 49]. In the present study, we identified a critical role for GC cell-derived TNF-α in promoting IL-33 expression in CAFs via a TNFR2/NF-κB/IRF-1 pathway. Cancer epithelial cells have highly effective ability to remold their stromal microenvironment, by exponentially amplifying cellular processes, such as inflammatory and wound healing, which further facilitates cancer progression [50, 51]. We herein addressed the pivotal function of TNF-α secreted from GC cells to synergistically promote the adjacent stromal cells to a pro-tumorigenic phenotype.

**Fig. 6:**
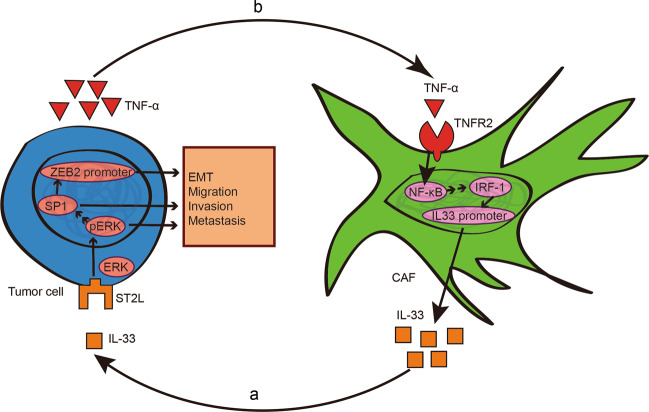
Model for the epithelial–stromal interactions in the tumor microenvironment of GC. **a** IL-33 released from CAFs promotes the migration, invasion and EMT of GC cells via the ST2L-ERK1/2-SP1-ZEB2 pathway. **b** GC cell-derived TNF-α upregulates IL-33 expression in CAFs via the TNFR2-NF-κB-IRF-1 pathway

Cross-talk between the epithelium and mesenchyme is considered to act as a functional driver of cancer development [11, 52, 53]. An intricate network of cross-talk between GC cells and CAFs favoring tumor progression is characterized in this study (Fig. 6). We have shown that IL-33 released by CAFs promotes the migration and invasion of GC cells via ST2L, which is dependent on the activation of the ERK1/2-SP1-ZEB2 pathway (Fig. 6a). Conversely, TNF-α is released by GC cells and induces IL-33 overexpression in CAFs via TNFR2-NF-κB-IRF-1 pathway (Fig. 6b). Thus, the cross-talk between CAFs and GC cells mediated by TNF-α/IL-33/ST2L signaling contributes to GC progression. Previous studies have demonstrated that IL-33 promotes cancer metastasis through recruitment of tumor-associated macrophages (TAMs) [54]. The immunological effects of IL-33 in GC progression will be further explored in our future studies.

In conclusion, we identified a complex crosstalk between CAFs and tumor cells in human GC leading to malignant phenotype, which is significantly associated with poor survival. Therefore, therapeutic intervention by interfering or targeting this positive feedback loop may benefit GC patient survival.

## Materials and methods

### Ethical statement

This study was approved by the Human Research Ethics Committee of Ruijin Hospital, Shanghai Jiao Tong University School of Medicine (Permit number: HREC08–028). Written informed consent was obtained from all the patients who were enrolled in this study. All animal experiments were approved by the Laboratory Animal Ethics Committee of Ruijin Hospital, Shanghai Jiao Tong University School of Medicine (Permit number: 2013062).

### Cell culture

SGC7901 and MKN45 cells were obtained from Beijing Cancer Hospital and the Cell Bank of Chinese Academy of Sciences, respectively, and cultured in RPMI-1640 medium containing 10% fetal bovine serum (no. 10500-064, GIBCO) with 100 U/ml penicillin and 100 U/ml streptomycin (no. 15140-122, GIBCO). The cell lines were authenticated by short tandem repeat analysis and had negative results for mycoplasma. Fibroblasts were isolated and cultured from GC tissues as described in our previous study [17]. Human recombinant IL-33 (no. AF-200-33) and TNF-α (no. AF-300-01A) were purchased from PeproTech. Anti-human IL-33 (no. AF3625), ST2L (no. AF523), TNF-α (no. AF-410-NA), TNFR1 (no. MAB225-100) and TNFR2 (no. MAB726-100) neutralizing antibodies were purchased from R&D Systems. ERK1/2 inhibitor U0126 (no. 9903) was from Cell Signaling Technology. Mithramycin A (no. ab142723) was purchased from Abcam. PDTC (no. P8765) and SN50 (no. HY-18738) were purchased from Sigma-Aldrich and MedchemExpress (Monmouth, NJ, USA).

### Tissue specimens and histological examination

A total of 134 GC tissues and matched normal tissues were collected from GC patients who underwent radical gastrectomy. None of these patients had received chemotherapy or radiation therapy before. All tissue samples were confirmed by two independent pathologists using standard methods. Thirty pairs of tissue samples were immediately imbedded in O.C.T. Compound (Sakura Finetek, USA) and frozen in −80 °C for cryosections. Eighteen pairs of tissue samples were frozen in liquid nitrogen for RNA extraction.

### Immunofluorescence

IF staining was performed as described previously [45]. Anti-IL-33 and anti-α-SMA antibodies were from Abcam. Anti-ST2L antibody was from Proteintech. Anti-FAP (no. sc-65398) and anti-Pan-Cytokeratin (no. sc-17843) antibodies were purchased from Santa Cruz. Anti-p65 (no. 8242) antibody was from Cell Signaling Technology. Images were obtained using an Olympus BX50 microscope (Olympus, Tokyo, Japan).

### Enzyme-linked immunosorbent assay

The protein levels of IL-33 or TNF-α in the supernatants of GC cells and CAFs were measured by human ELISA kit (eBioscience, CA, USA) according to the manufacturer’s protocols.

### IHC staining

IHC was performed as described previously [45]. The slides were assessed by two independent pathologists who were blinded to clinicopathologic information. The percentage of positive cells was classified into four grades (percentage scores): 0% (0), 1–30% (1), 31–60% (2), 61–100% (3). The staining intensity was classified into four grades (intensity scores): no staining (0), light brown staining (1), brown staining (2), dark brown staining (3). We multiplied the percentage scores and intensity scores to get total scores: weak positive (0–3), strong positive (≥4).

### Western blot analysis

Immunoblots of GC cells were performed as described previously [45]. Anti-GADPH (no. HRP-60004), anti-α-cantenin (no. 12831-1-AP), anti-E-cadherin (no. 20874-1-AP), anti-N-cadherin (no. 22018-1-AP), anti-ZEB2 (no. ab138222), anti-ST2L (no.11920-1-AP), and anti-SP1 (no. 21962-1-AP) antibodies were purchased from Proteintech. Anti ERK1/2 (no. 9100S), anti-pERK1/2 (no. 9100S) and HRP-conjugated secondary antibodies were purchased from Cell Signaling Biotechnology.

### QRT-PCR

QRT-PCR was performed to detect the mRNA levels of IL-33, TNF-α, ST2L, IRF-1, E-cadherin, N-cadherin, α-catenin, SP1 and ZEB2 as described previously [17]. GAPDH was used as the internal control. The sequences of primers used in this study are shown in Table S1.

### RNA interference

Human IL-33 siRNA (no. sc-75333, Santa Cruz), ST2L siRNA (Biotend), IRF-1 siRNA (GenePharma) or scrambled siRNA was transfected into CAFs or GC cells with Lipofectamine 2000 reagent (Invitrogen, Carlsbad, CA, USA). Supernatants and cells were collected within 24 h after transfection for further experiments.

### Plasmid construction and transfection

Human IRF-1, SP1 overexpression plasmids and control plasmids were constructed and transfected into CAFs or GC cells using Lipofectamine 2000 reagent (Invitrogen, Carlsbad, CA, USA) according to the manufacturer’s instructions.

### Generation of ST2L knockout cell lines by CRISPR/Cas9 system

Five ST2L sgRNAs were synthesized and cloned into lentiCRISPR.V2 plasmid (Addgene, #52961) respectively. GC cells were infected by lentiviral supernatants and ST2L expression was validated by western blot. The fifth sgRNA (GGCACACCGTAAGACTAAGT) was the most effective in GC cells.

### Cell migration and invasion assays

Cell migration and invasion assays were performed as described previously [17]. Nine fields were randomly selected to count the average number of migrated cells.

### Luciferase reporter assays

Human IL-33 or ZEB2 promoters were amplified and cloned into pGL3 basic vector containing the firefly luciferase reporter gene. Firefly and Renilla luciferase activities were detected at 48 h after transfection using the Dual-Luciferase Reporter Assay System (Promega, Madison, WI, USA).

### In vivo peritoneal dissemination

Male BALB/c nude mice at the age of 28 days were purchased from the Institute of Zoology, Chinese Academy of Sciences, and housed in a specific pathogen-free environment. Mice were injected intraperitoneally with GC cells or the combination of GC cells and CAFs (*n* = 5 per group). All mice were sacrificed under anesthesia at 45 days after injection. Laparotomy was performed to observe metastasis in the peritoneal cavity.

### Statistical analysis

All experiments were repeated at least three times and results were displayed as means ± standard deviation (SD). Differences in the frequency of IL-33 and ST2L expression and the association with clinicopathological characteristics were statistically analyzed using the Pearson *χ*^2^ test. Differences between parallel experimental groups were evaluated using the Student’s *t* test and one-way ANOVA. A value of *P* < 0.05 (two-tailed) was considered as statistically significant. All statistical analyses were conducted using the software Stata 12.0 (Stata Corporation, College Station, TX, USA).

## Supplementary information

Supplemental Figure Legends

Supplemental Table S1

Supplemental Figure S1

Supplemental Figure S2

Supplemental Figure S3

Supplemental Figure S4

Supplemental Figure S5

Supplemental Figure S6

Supplemental Figure S7

## References

[1] Kamangar F, Dores GM, Anderson WF (2006). Patterns of cancer incidence, mortality, and prevalence across five continents: defining priorities to reduce cancer disparities in different geographic regions of the world. J Clin Oncol..

[2] Jemal A, Bray F, Center MM, Ferlay J, Ward E, Forman D (2011). Global cancer statistics. CA Cancer J Clin..

[3] Ajani JA, Lee J, Sano T, Janjigian YY, Fan D, Song S (2017). Gastric adenocarcinoma. Nat Rev Dis Prim..

[4] Lordick F, Shitara K, Janjigian YY (2017). New agents on the horizon in gastric cancer. Ann Oncol..

[5] Bray F, Ferlay J, Soerjomataram I, Siegel RL, Torre LA, Jemal A (2018). Global cancer statistics 2018: GLOBOCAN estimates of incidence and mortality worldwide for 36 cancers in 185 countries. CA Cancer J Clin..

[6] Tsujimoto H, Ono S, Ichikura T, Matsumoto Y, Yamamoto J, Hase K (2010). Roles of inflammatory cytokines in the progression of gastric cancer: friends or foes?. Gastric Cancer..

[7] Chia NY, Tan P (2016). Molecular classification of gastric cancer. Ann Oncol..

[8] Bhowmick NANE, Moses HL (2004). Stromal fibroblasts in cancer initiation and progression. Nature..

[9] Bhowmick NAMH (2005). Tumor-stroma interactions. Curr Opin Genet Dev.

[10] Xing F, Saidou J, Watabe K (2010). Cancer associated fibroblasts (CAFs) in tumor microenvironment. Front Biosci..

[11] Liotta LA, Kohn EC (2001). The microenvironment of the tumour-host interface. Nature..

[12] Gascard P, Tlsty TD (2016). Carcinoma-associated fibroblasts: orchestrating the composition of malignancy. Genes Dev..

[13] Labernadie A, Kato T, Brugues A, Serra-Picamal X, Derzsi S, Arwert E (2017). A mechanically active heterotypic E-cadherin/N-cadherin adhesion enables fibroblasts to drive cancer cell invasion. Nat Cell Biol..

[14] Cirri P, Chiarugi P (2012). Cancer-associated-fibroblasts and tumour cells: a diabolic liaison driving cancer progression. Cancer Metastasis Rev..

[15] Ridge SM, Sullivan FJ, Glynn SA (2017). Mesenchymal stem cells: key players in cancer progression. Mol Cancer..

[16] Glentis A, Oertle P, Mariani P, Chikina A, El Marjou F, Attieh Y (2017). Cancer-associated fibroblasts induce metalloprotease-independent cancer cell invasion of the basement membrane. Nat Commun..

[17] Wu X, Chen X, Zhou Q, Li P, Yu B, Li J (2013). Hepatocyte growth factor activates tumor stromal fibroblasts to promote tumorigenesis in gastric cancer. Cancer Lett..

[18] Kakkar R, Hei H, Dobner S, Lee RT (2012). Interleukin 33 as a mechanically responsive cytokine secreted by living cells. J Biol Chem..

[19] Schmitz J, Owyang A, Oldham E, Song Y, Murphy E, McClanahan TK (2005). IL-33, an interleukin-1-like cytokine that signals via the IL-1 receptor-related protein ST2 and induces T helper type 2-associated cytokines. Immunity..

[20] Liew FY, Girard JP, Turnquist HR (2016). Interleukin-33 in health and disease. Nat Rev Immunol..

[21] Oboki K, Ohno T, Kajiwara N, Saito H, Nakae S (2010). IL-33 and IL-33 receptors in host defense and diseases. Allergol Int..

[22] Coyle AJ, Lloyd C, Tian J, Nguyen T, Erikkson C, Wang L (1999). Crucial role of the interleukin 1 receptor family member T1/ST2 in T helper cell type 2-mediated lung mucosal immune responses. J Exp Med..

[23] Townsend MJ, Fallon PG, Matthews DJ, Jolin HE, McKenzie AN (2000). T1/ST2-deficient mice demonstrate the importance of T1/ST2 in developing primary T helper cell type 2 responses. J Exp Med..

[24] Carriere V, Roussel L, Ortega N, Lacorre DA, Americh L, Aguilar L (2007). IL-33, the IL-1-like cytokine ligand for ST2 receptor, is a chromatin-associated nuclear factor in vivo. Proc Natl Acad Sci USA..

[25] Gramatzki D, Frei K, Cathomas G, Moch H, Weller M, Mertz KD (2016). Interleukin-33 in human gliomas: expression and prognostic significance. Oncol Lett..

[26] Yang ZP, Ling DY, Xie YH, Wu WX, Li JR, Jiang J (2015). The association of serum IL-33 and sST2 with breast cancer. Dis Markers..

[27] Tong X, Barbour M, Hou K, Gao C, Cao S, Zheng J (2016). Interleukin-33 predicts poor prognosis and promotes ovarian cancer cell growth and metastasis through regulating ERK and JNK signaling pathways. Mol Oncol..

[28] Zeng X, Zhang Z, Gao QQ, Wang YY, Yu XZ, Zhou B (2016). Clinical significance of serum interleukin-31 and interleukin-33 levels in patients of endometrial cancer: a case control study. Dis Markers..

[29] Ye XL, Zhao YR, Weng GB, Chen YC, Wei XN, Shao JP (2015). IL-33-induced JNK pathway activation confers gastric cancer chemotherapy resistance. Oncol Rep..

[30] Levescot A, Flamant S, Basbous S, Jacomet F, Feraud O, Anne Bourgeois E (2014). BCR-ABL-induced deregulation of the IL-33/ST2 pathway in CD34+ progenitors from chronic myeloid leukemia patients. Cancer Res..

[31] Cayrol C, Girard JP (2018). Interleukin-33 (IL-33): a nuclear cytokine from the IL-1 family. Immunol Rev..

[32] Li J, Razumilava N, Gores GJ, Walters S, Mizuochi T, Mourya R (2014). Biliary repair and carcinogenesis are mediated by IL-33-dependent cholangiocyte proliferation. J Clin Invest..

[33] Schmieder A, Multhoff G, Radons J (2012). Interleukin-33 acts as a pro-inflammatory cytokine and modulates its receptor gene expression in highly metastatic human pancreatic carcinoma cells. Cytokine..

[34] Sun P, Ben Q, Tu S, Dong W, Qi X, Wu Y (2011). Serum interleukin-33 levels in patients with gastric cancer. Dig Dis Sci..

[35] Larsen KM, Minaya MK, Vaish V, Pena MMO. The role of IL-33/ST2 pathway in tumorigenesis. Int J Mol Sci. 2018;19:2676. 10.3390/ijms19092676 PMC6164146 30205617

[36] Maywald RL, Doerner SK, Pastorelli L, De Salvo C, Benton SM, Dawson EP (2015). IL-33 activates tumor stroma to promote intestinal polyposis. Proc Natl Acad Sci USA..

[37] Chang KW, Huang YL, Wong ZR, Su PH, Huang BM, Ju TK (2013). Fibroblast growth factor-2 up-regulates the expression of nestin through the Ras-Raf-ERK-Sp1 signaling axis in C6 glioma cells. Biochem Biophys Res Commun..

[38] Lin T, Chen Y, Ding Z, Luo G, Liu J, Shen J (2013). Novel insights into the synergistic interaction of a thioredoxin reductase inhibitor and TRAIL: the activation of the ASK1-ERK-Sp1 pathway. PLoS ONE..

[39] Nam EH, Lee Y, Zhao XF, Park YK, Lee JW, Kim S (2014). ZEB2-Sp1 cooperation induces invasion by upregulating cadherin-11 and integrin alpha5 expression. Carcinogenesis..

[40] Zhang BG, Du T, Zang MD, Chang Q, Fan ZY, Li JF (2014). Androgen receptor promotes gastric cancer cell migration and invasion via AKT-phosphorylation dependent upregulation of matrix metalloproteinase 9. Oncotarget..

[41] Yang S, Wang Y, Mei K, Zhang S, Sun X, Ren F (2015). Tumor necrosis factor receptor 2 (TNFR2).interleukin-17 receptor D (IL-17RD) heteromerization reveals a novel mechanism for NF-kappaB activation. J Biol Chem..

[42] Cirri P, Chiarugi P (2013). Tumors and their stroma: mitochondria at the crossroad. Cell Cycle..

[43] Andersson P, Yang Y, Hosaka K, Zhang Y, Fischer C, Braun H, et al. Molecular mechanisms of IL-33-mediated stromal interactions in cancer metastasis. JCI Insight. 2018;3:122375. 10.1172/jci.insight.122375 PMC6237443 30333314

[44] Chen SF, Nieh S, Jao SW, Wu MZ, Liu CL, Chang YC (2013). The paracrine effect of cancer-associated fibroblast-induced interleukin-33 regulates the invasiveness of head and neck squamous cell carcinoma. J Pathol..

[45] Wang X, Zhou Q, Yu Z, Wu X, Chen X, Li J (2017). Cancer-associated fibroblast-derived Lumican promotes gastric cancer progression via the integrin beta1-FAK signaling pathway. Int J Cancer..

[46] Wu X, Tao P, Zhou Q, Li J, Yu Z, Wang X (2017). IL-6 secreted by cancer-associated fibroblasts promotes epithelial-mesenchymal transition and metastasis of gastric cancer via JAK2/STAT3 signaling pathway. Oncotarget..

[47] Kasagi Y, Harada Y, Morodomi Y, Iwai T, Saito S, Yoshida K (2016). Peritoneal dissemination requires an Sp1-dependent CXCR4/CXCL12 signaling axis and extracellular matrix-directed spheroid formation. Cancer Res..

[48] Erez N, Truitt M, Olson P, Arron ST, Hanahan D (2010). Cancer-associated fibroblasts are activated in incipient neoplasia to orchestrate tumor-promoting inflammation in an NF-kappaB-dependent manner. Cancer Cell..

[49] Akimoto M, Hayashi JI, Nakae S, Saito H, Takenaga K (2016). Interleukin-33 enhances programmed oncosis of ST2L-positive low-metastatic cells in the tumour microenvironment of lung cancer. Cell Death Dis..

[50] Fukumura D, Xavier R, Sugiura T, Chen Y, Park EC, Lu N (1998). Tumor induction of VEGF promoter activity in stromal cells. Cell..

[51] Schauer IG, Sood AK, Mok S, Liu J (2011). Cancer-associated fibroblasts and their putative role in potentiating the initiation and development of epithelial ovarian cancer. Neoplasia..

[52] Lu M, Jolly MK, Levine H, Onuchic JN, Ben-Jacob E (2013). MicroRNA-based regulation of epithelial-hybrid-mesenchymal fate determination. Proc Natl Acad Sci USA..

[53] Avgustinova A, Iravani M, Robertson D, Fearns A, Gao Q, Klingbeil P (2016). Tumour cell-derived Wnt7a recruits and activates fibroblasts to promote tumour aggressiveness. Nat Commun..

[54] Yang Y, Andersson P, Hosaka K, Zhang Y, Cao R, Iwamoto H (2016). The PDGF-BB-SOX7 axis-modulated IL-33 in pericytes and stromal cells promotes metastasis through tumour-associated macrophages. Nat Commun..

