# Supplementary material for: The reciprocal interaction between tumor cells and activated fibroblasts mediated by TNF-α/IL-33/ST2L signaling promotes gastric cancer metastasis
Source: Oncogene. 2019 Oct 28;39(7):1414–28. doi: 10.1038/s41388-019-1078-x (PMC7018661; doi:10.1038/s41388-019-1078-x)
Supplement: Supplementary file 1 — Supplemental Figure Legends [file 41388_2019_1078_MOESM1_ESM.docx]

**Supplemental Figure Legends**

**Figure S1. The mRNA and protein levels of *IL-33* and ST2L in human GC cell lines, CAFs and NFs. (a)** The mRNA levels of *IL-33* in human GC cell lines, CAFs and NFs were measured by QRT-PCR. **(b)** The protein levels of IL-33 in human GC cell lines, CAFs and NFs were measured by ELISA. **(c)** The protein levels of ST2L in human GC cell lines, CAFs and NFs were measured by Western blot analysis.

**Figure S2. Knockdown of IL-33 expression by RNAi. (a)** *IL-33* mRNA levels were measured by QRT-PCR in CAFs transfected with control siRNA (siRNA/NC) or IL-33 targeting siRNA (siRNA/IL-33). **(b)** IL-33 protein levels were measured by ELISA in CAFs transfected with control siRNA (siRNA/NC) or IL-33 targeting siRNA (siRNA/IL-33). Values represent means ± SD (n = 3). (*P<0.05).

**Figure S3. The mRNA and protein levels of SP1 in GC cell lines, NFs and CAFs. (a)** The mRNA levels of *SP1* in GC cell lines, NFs and CAFs were measured by QRT-PCR. **(b)** The protein levels of SP1 in GC cell lines, NFs and CAFs were measured by Western blot. **(c)**The binding sites of SP1 in ZEB2 promoter region (all sites were highlighted in blue and green). **(d)** The binding efficiency were evaluated using two online tools (*http://jaspar.genereg.net;* *http://alggen.lsi.upc.es/cgi-bin/promo_v3/promo/promomenu.cgi?dirDB=TF_8.3&Option=3&idCon=155533369100*).

**Figure S4. Knockdown of SP1 or ZEB2 expression reverses EMT of GC cells induced by CAF-derived IL-33.** The protein levels of EMT markers in SGC7901 cells **(a) or** MKN45 cells **(b)** were detected after incubation with the following stimuli: exogenous IL-33 (300 ng/ml); SP1 inhibitor (Mithramycin A: 5 μM); transfection with ZEB2 siRNA (**c**) and (**d**); or co-culture with CAFs in the presence of SP1 inhibitor.

**Figure S5. The mRNA levels of *TNF-α* in human GC tissues and cell lines. (a)** The mRNA levels of *TNF-α* in human GC tissues (n=30) were measured by QRT-PCR. **(b)** The mRNA levels of *TNF-α* in human GC cell lines, GES-1, NFs and CAFs were measured by QRT-PCR. (**c**)The mRNA levels of *TNF-α* in human GC cell lines and GES-1 were measured by QRT-PCR. (d) The IRF-1-binding sites in the IL-33 promoter region as predicted by JASPAR on-line tool ([*http://jaspar.genereg.net*)](http://jaspar.genereg.net)).

**Figure S6. The nuclear translocation of NF-κB p65 in CAFs.** The nuclear translocation of NF-κB p65 in CAFs was detected by IF after culture in medium alone or activation with the following stimuli: exogenous TNF-α (50 ng/ml); supernatants from SGC7901cells (SGC7901su) or from MKN45 cells (MKN45su); SN50 (5 μM); PDTC (5 μM); IgG isotype antibody (3 μg/ml); anti-TNFR1 (10 μg/ml) or anti-TNFR2 (10 μg/ml) neutralizing antibodies.

**Figure S7. The expression of IL-33 and ZEB2 in peritoneal nodules.** Immunohistochemistry analyses of IL-33 and ZEB2 protein expression in peritoneal nodules from GC cells mixed with CAFs-siRNA/NC or CAFs-siRNA/IL-33.
