# Supplementary material for: The reciprocal interaction between tumor cells and activated fibroblasts mediated by TNF-α/IL-33/ST2L signaling promotes gastric cancer metastasis
Source: Oncogene. 2019 Oct 28;39(7):1414–28. doi: 10.1038/s41388-019-1078-x (PMC7018661; doi:10.1038/s41388-019-1078-x)
Supplement: Supplementary file 2 — Supplemental Table S1 [file 41388_2019_1078_MOESM2_ESM.docx]

**Table S1. The sequences of primers used in this study.**

| **Gene name** | **Sequence (5'->3')** | **Number of bases (bp)** | **Annealing Temperature (℃)** | **Amplicon Size (bp)** |
| --- | --- | --- | --- | --- |
| IL33-F | CCTGTCAACAGCAGTCTAC | 19 | 60 | 340 |
| IL33-R | TTGGCATGCAACCAGAAGTC | 20 | 60 |  |
| TNFR1-F | ATGTTAAGGGCACTGAGGACT | 21 | 60 | 199 |
| TNFR1-R | AAGAAAATGACCAGGGGCAAC | 21 | 60 |  |
| TNFR2-F | TGTAGCCAAGGTCGGTAAGTT | 21 | 60 | 245 |
| TNFR2-R | CAATTTATCACCCTGCCCCTG | 21 | 60 |  |
| IRF-1-F | ATGCCCATCACTCGGATGC | 19 | 60 | 204 |
| IRF-1-R | CCCTGCTTTGTATCGGCCTG | 20 | 60 |  |
| TNF-α-F | TCTTCTCGAACCCCGAGTGA | 20 | 60 | 151 |
| TNF-α-R | CCTCTGATGGCACCACCAG | 19 | 60 |  |
| E-cadherin-F | ATTTTTCCCTCGACACCCGAT | 21 | 60 | 109 |
| E-cadherin-R | TCCCAGGCGTAGACCAAGA | 19 | 60 |  |
| α-catenin-F | GGGGATAAAATTGCGAAGGAGA | 22 | 60 | 144 |
| α-catenin-R | GTTGCCTCGCTTCACAGAAGA | 21 | 60 |  |
| N-cadherin-F | AGCCAACCTTAACTGAGGAGT | 21 | 60 | 136 |
| N-cadherin-R | GGCAAGTTGATTGGAGGGATG | 21 | 60 |  |
| ZEB2-F | GCGATGGTCATGCAGTCAG | 19 | 60 | 138 |
| ZEB2-R | CAGGTGGCAGGTCATTTTCTT | 20 | 60 |  |
| SP1-F | GAGCAAAACCAGCAGACACA | 20 | 60 | 203 |
| SP1-R | ACTGTTGGTGTCCGGATGAT | 20 | 60 |  |
| ST2L-F | CAGAGAGAGGCACAACAGGA | 20 | 60 | 214 |
| ST2L-R | AGACCCACAGAACACTACGG | 20 | 60 |  |
| GAPDH-F | GGACCTGACCTGCCGTCTAG | 20 | 60 | 100 |
| GAPDH-R | GTAGCCCAGGATGCCCTTGA | 20 | 60 |  |

F: Forward, R: Reverse
