# Supplementary figures and images for: The reciprocal interaction between tumor cells and activated fibroblasts mediated by TNF-α/IL-33/ST2L signaling promotes gastric cancer metastasis
Source: Oncogene. 2019 Oct 28;39(7):1414–28. doi: 10.1038/s41388-019-1078-x (PMC7018661; doi:10.1038/s41388-019-1078-x)

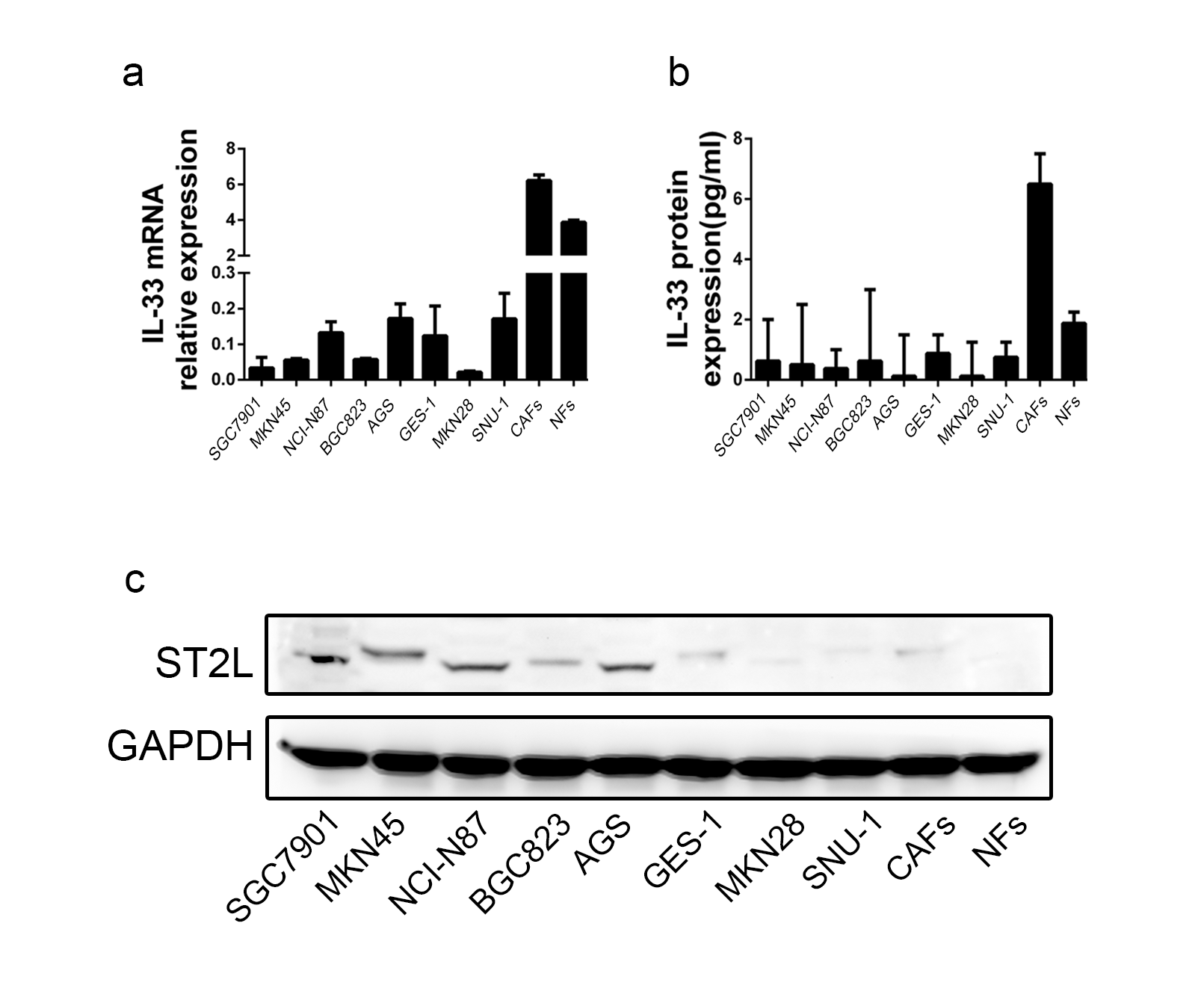

Supplement: Supplementary file 3 — Supplemental Figure S1 [file 41388_2019_1078_MOESM3_ESM.tif]

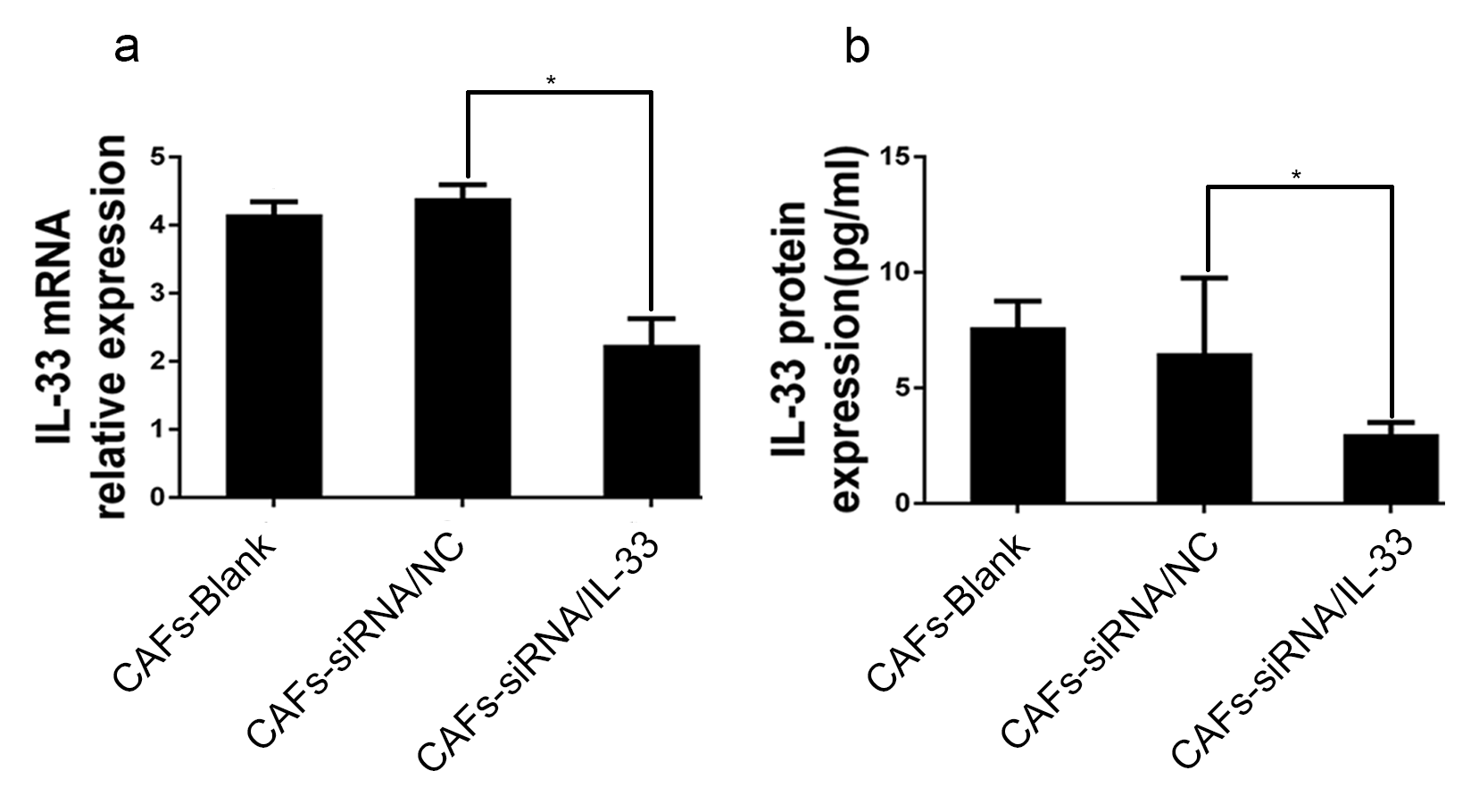

Supplement: Supplementary file 4 — Supplemental Figure S2 [file 41388_2019_1078_MOESM4_ESM.tif]

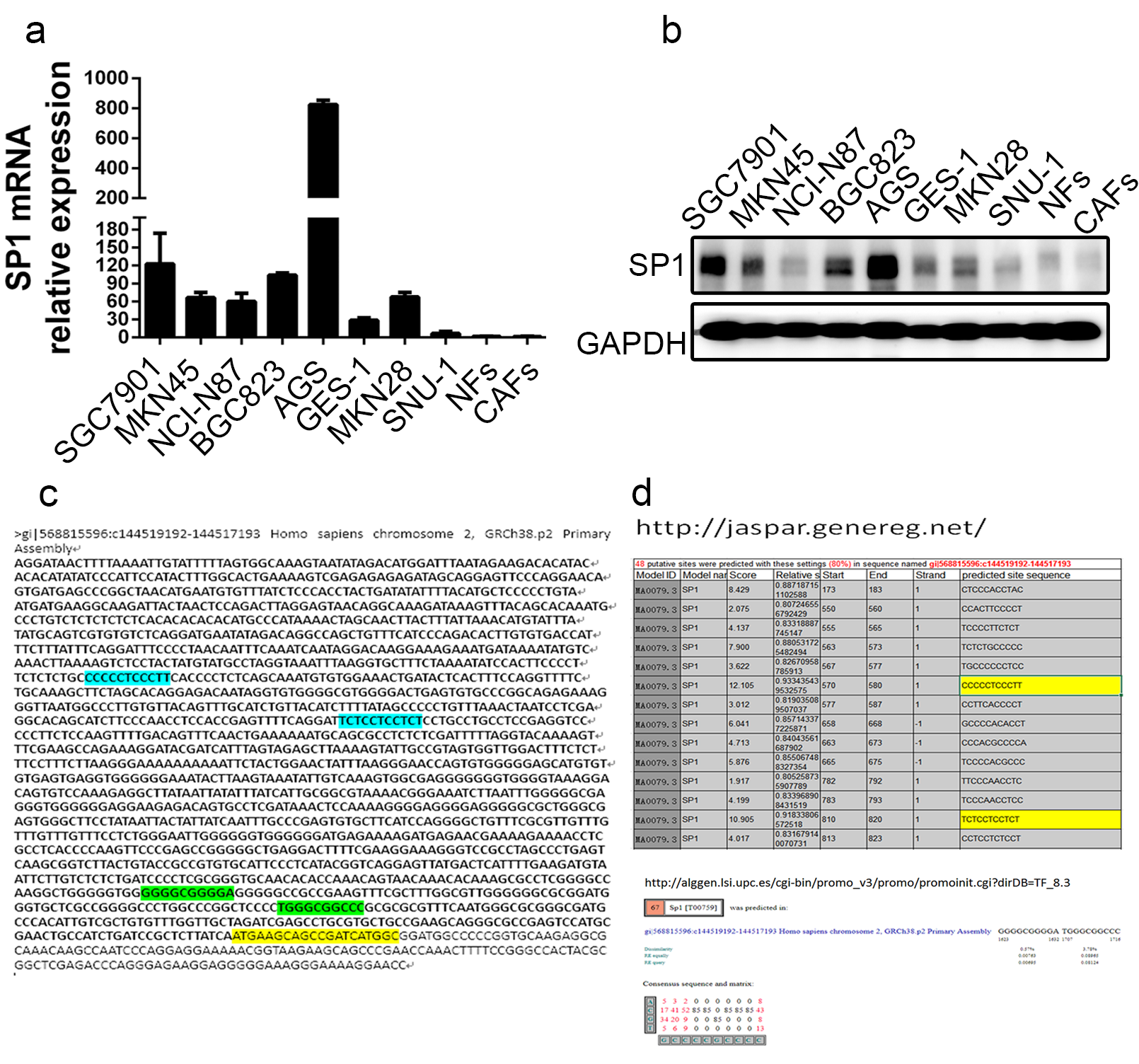

Supplement: Supplementary file 5 — Supplemental Figure S3 [file 41388_2019_1078_MOESM5_ESM.tif]

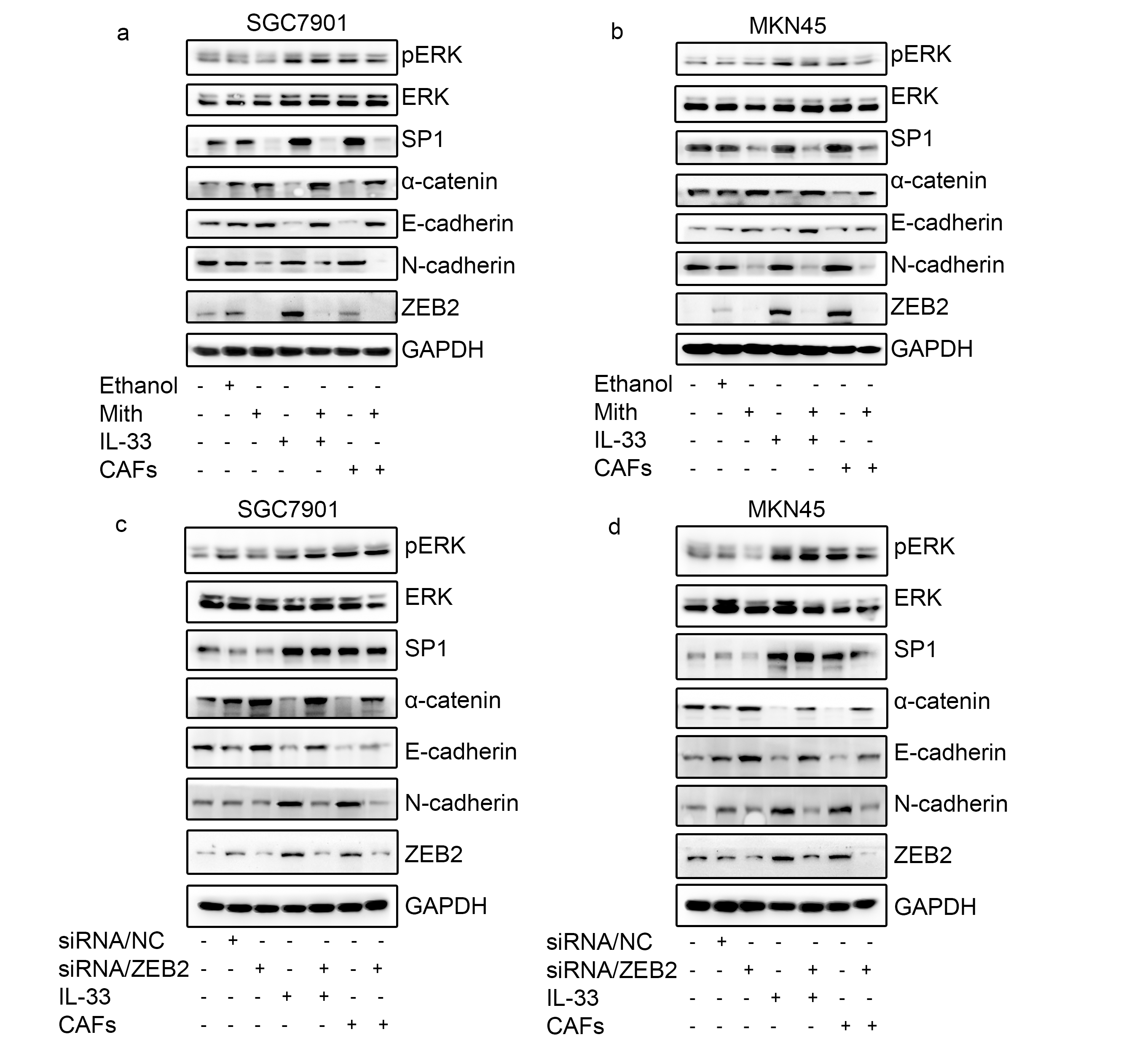

Supplement: Supplementary file 6 — Supplemental Figure S4 [file 41388_2019_1078_MOESM6_ESM.tif]

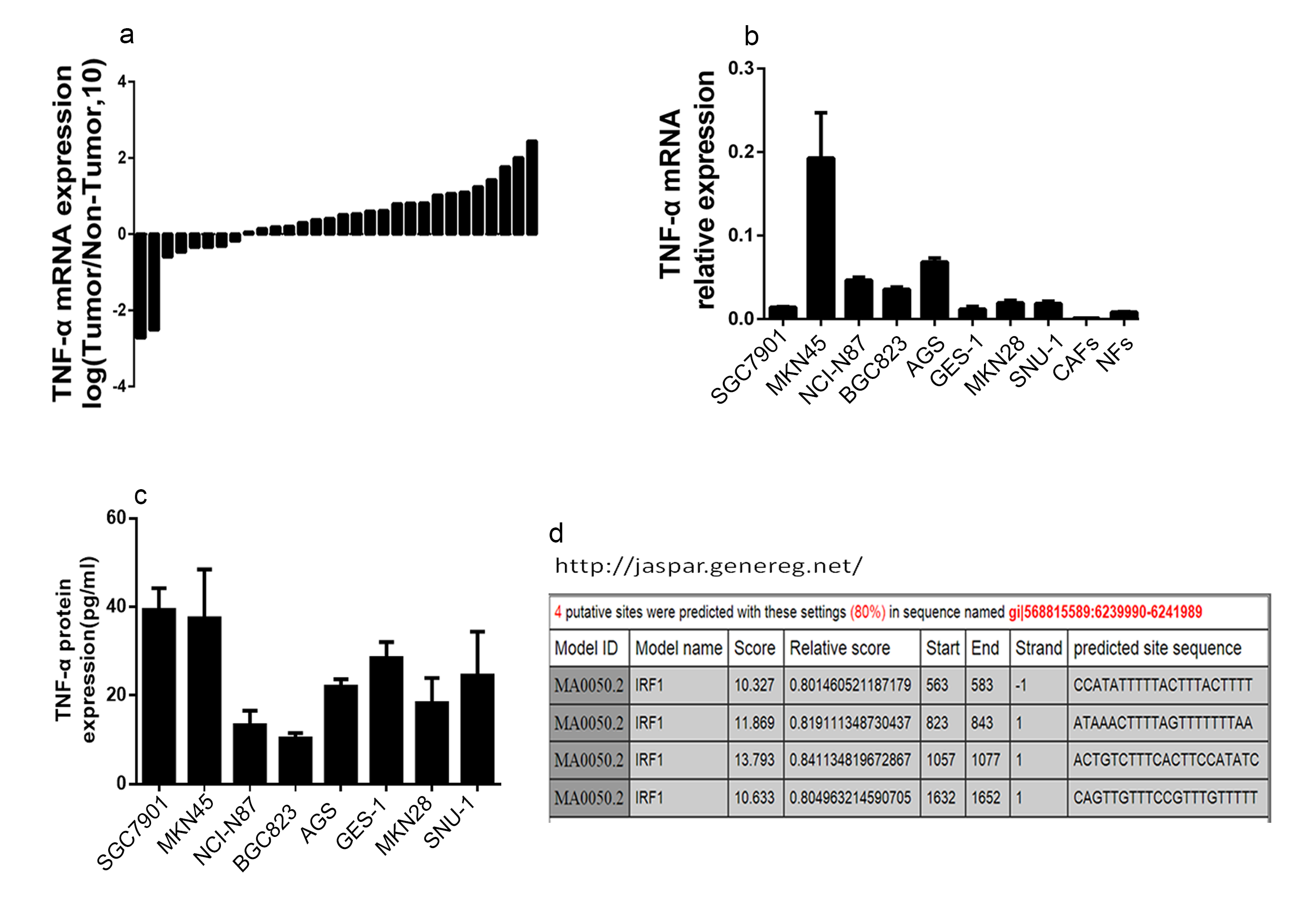

Supplement: Supplementary file 7 — Supplemental Figure S5 [file 41388_2019_1078_MOESM7_ESM.tif]

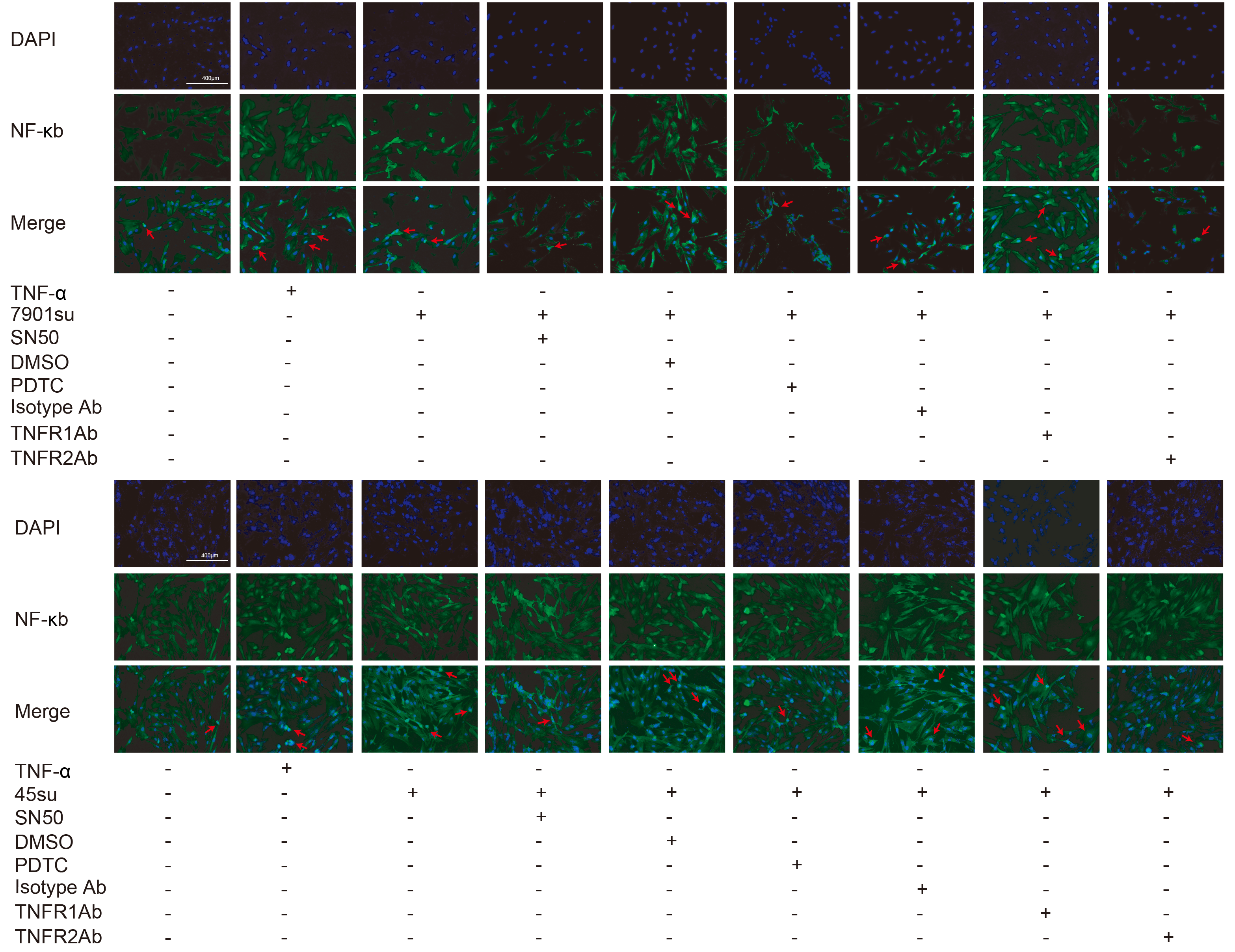

Supplement: Supplementary file 8 — Supplemental Figure S6 [file 41388_2019_1078_MOESM8_ESM.tif]

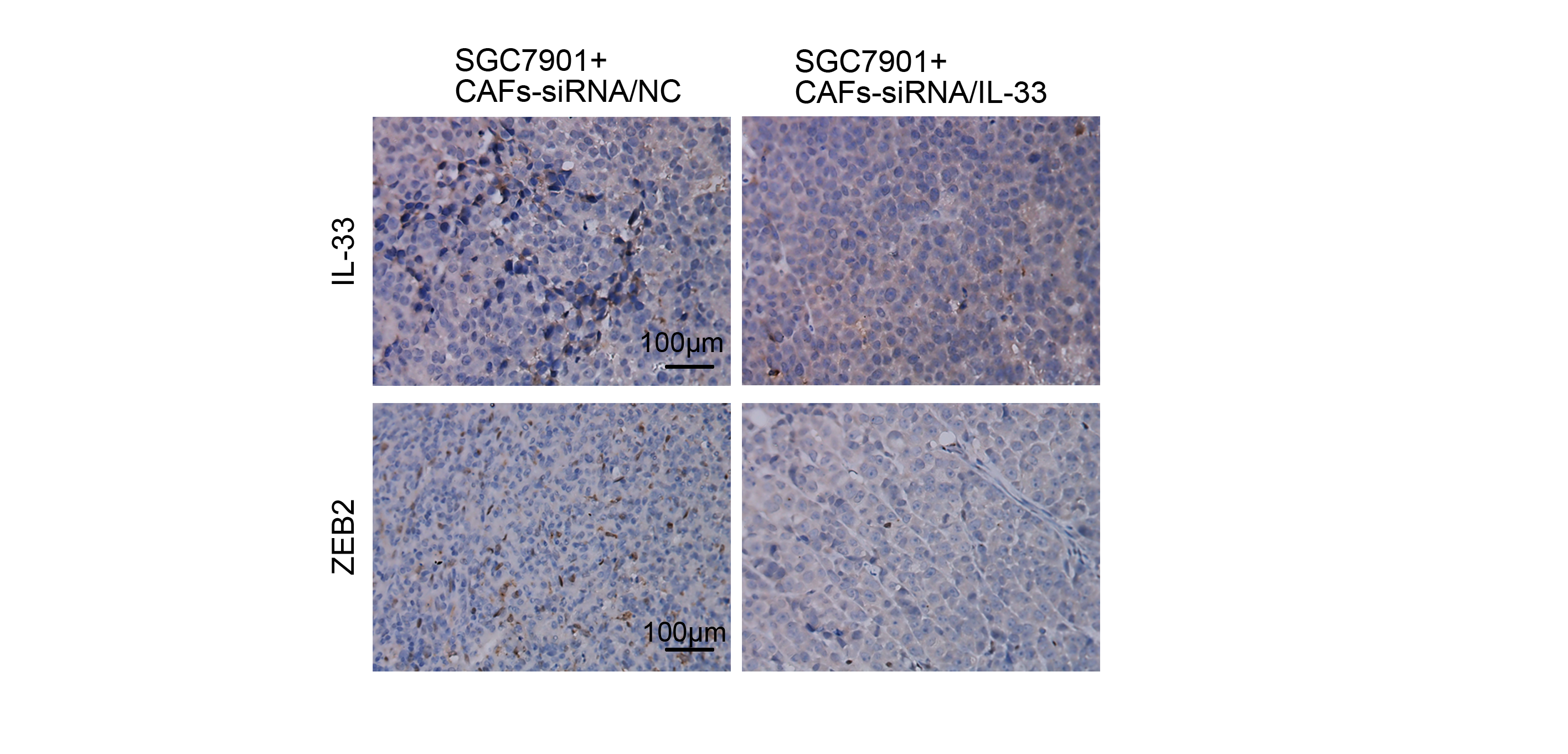

Supplement: Supplementary file 9 — Supplemental Figure S7 [file 41388_2019_1078_MOESM9_ESM.tif]
